# Oculomotor Training Improves Reading and Associated Cognitive Functions in Children with Learning Difficulties: A Pilot Study

**DOI:** 10.3390/vision9040083

**Published:** 2025-10-07

**Authors:** Alessio Facchin, Silvio Maffioletti, Marta Maffioletti, Gabriele Esposito, Marta Bonetti, Luisa Girelli, Roberta Daini

**Affiliations:** 1 Department of Human and Social Sciences, Mercatorum University, 00186 Rome, Italy; 2 Department of Psychology, University of Milano-Bicocca, 20126 Milan, Italy; luisa.girelli@unimib.it (L.G.); roberta.daini@unimib.it (R.D.); 3 Institute of Research and Studies in Optics and Optometry, 50059 Vinci, Italy; silvio.maffioletti@gmail.com; 4 Degree Course in Optics and Optometry, University of Torino, 10124 Torino, Italy; 5 Leonardo da Vinci Study Center, 24127 Bergamo, Italy; maffiomarta@gmail.com; 6 Prospettiva Medical Center, 24124 Bergamo, Italy; gabriele_esposito@icloud.com (G.E.); bonettimarta@gmail.com (M.B.); 7 Civic Institute of Optometry, 20157 Milano, Italy; 8 IRCCS Fondazione Don Carlo Gnocchi ONLUS, 20126 Milan, Italy; 9 COMiB—Optics and Optometry Research Center, University of Milano-Bicocca, 20126 Milan, Italy

**Keywords:** reading, eye movements, oculomotor training, DEM test, saccades, reading difficulties, dyslexia

## Abstract

In the first years of schooling, inefficient eye movements can impair the development of reading skills. Nonetheless, the improvement of these abilities has been little investigated in children. This pilot study aimed to verify the effectiveness of Office Based Oculomotor Training (OBOT) in enhancing reading skills in ‘poor’ readers. Twenty-one children (aged 7–12 years) underwent an assessment of reading, visual, and perceptual abilities before and after a training of oculomotor skills (i.e., execution of saccadic movements with symbol charts in various modes and types; 14 participants) or a simple reading exercise (7 participants). The overall duration of the training was six weeks. The results showed a specific improvement, in the group subjected to oculomotor training only, not only in oculomotor abilities but also in reading, visuo-perceptual skills, and the ability to resolve crowding. These primary results suggest that the improvement of oculomotor abilities can lead to an indirect increase in reading in developmental age.

## 1. Introduction

Reading acquisition is a complex process, which involves many different aspects and is closely intertwined with the development of a child’s language skills [1]. Learning to read involves the process of adapting to seeing the shapes and forms associated with the letters in the different alphabets [2]. It has been reported that 73% of children who were at risk of reading problems were experiencing language milestone delays in preschool [3]. Reading is a complex skill that involves many cognitive functions, visual perception and attention, language, motor programming, and execution. Therefore, acquiring reading skills is preceded by the development and mastery of those underlying skills and, successively, their integration [4]. Consequently, it is not surprising the high incidence/prevalence of children with reading deficits [5].

Nevertheless, learning to read may be a lengthy and effortful process also for children whose difficulties, overall, do not satisfy the criteria for a clinical diagnosis of specific learning disorders (SLD). These difficulties may impact various parameters such as speed of reading, accuracy, fluency, comprehension, and phonetics. They are often referred to as “other reading problems” or “learning difficulties” [6]. It is important to recognize and respond to these difficulties, as they can indeed have a significant impact on a child’s overall efficiency in facing the requests of schooling. Moreover, since these difficulties are, by definition, mild, they can be prone to improvement if subjected to specific treatments, eventually resulting in an effective full normalization of the reading performance.

Vision plays a crucial role in the acquisition of reading skills. There are different aspects that link reading skills and vision. The first central aspect is known as crowding [7]. As the name suggests, crowding can be defined as the difficulty in recognizing objects when they are surrounded by similar items. During reading, excessive crowding can lead to the inability to recognize letters when they are surrounded by other letters [8]. The result of this problem is a slower reading speed and a greater tendency to make errors while reading. Excessive visual crowding is often associated with developmental dyslexia (DD) [7]. Studies have shown that processes that contrast crowding (e.g., focal attention) develop after a child learns to read [9].

In addition, peripheral vision problems can interfere with reading. There are several pieces of evidence that the prevalence of visual deficits is more common in children with specific learning disorders than in age-matched controls [10,11,12]. One of these deficits concerns oculomotor efficiency disorders. Reading requires accurate eye movement control to explore written text. Good readers usually perform only accurate saccades during reading [13]. Poor readers, on the other hand, tend to make different degrees of inaccurate saccades, finally resulting in a slower reading speed [14,15,16,17]. In addition, they may miss words or even entire lines [18]. This can lead to difficulty understanding the content, making it harder to learn and remember, with the need to re-explore the text to read it. For example, abnormal oculomotor control was reported in dyslexic children, characterized by a longer fixation time and a greater number of fixations, as well as more and shorter saccades [19,20,21,22,23,24] and also lower gain in pursuit eye movements [25]. These oculomotor deficits were found in populations of different languages in poor readers [26,27,28].

Yet, with respect to other factors contributing to reading difficulties, such as phonological processes deficits, attentional deficits, and language disorders [29,30,31,32], oculomotor deficits have been so far very rarely targeted to specific treatments. As an exception, an oculomotor training was developed by Bucci and colleagues [33]. The training consisted of four specific tasks: (i) rapid naming, (ii) Stroop task, (iii) motion perception, and (iv) Saccadic eye movement training. The training duration was 15 min a day, 5 days a week, for 8 weeks. The sixteen dyslexic Italian children participants improved reading time and fixation time. Despite the improvement, the contribution of the exercises that specifically concerned eye movements cannot be separated from the whole treatment.

Another study on the effect of an oculomotor enhancement program was performed by Jafarlou et al. [21]. Thirty dyslexic children from 8 to 12 years with oculomotor deficits were trained with eye movement exercises for gaze fixation, saccades, and pursuits while the control group received no intervention. Results showed improvements in most of the eye movement parameters, and, interestingly, also positive effects on visual discrimination and flexibility of attention, visual sustained attention, and spatial working memory [34]. Other than developmental dyslexia, oculomotor training was also applied in other conditions and populations. One of the first pioneering studies examined the relationship between oculomotor behaviour and complex perceptual motor skill acquisition, testing whether inefficient oculomotor behaviour can be improved [35]. Results showed that oculomotor efficiency correlates with measures of game performance, used to assess complex visuomotor tasks. More, oculomotor inefficiency can be remedied with specific eye movement computerized training organised into different levels. Finally, the patterns of efficient eye movements trained by oculomotor exercises are transferred to the complex perceptual motor tasks involved in gaming [35].

A study from the same group has investigated the effects of oculomotor rehabilitation in 30 children (15 treatment and 15 control group) with dyslexia who exhibited oculomotor abnormalities. The intervention consisted of an 8-week oculomotor training program targeting saccades, tracking, and fixation, combined with home-based exercises. Reading abilities and eye movement parameters were assessed both before and after the intervention. Results showed that children in the treatment group exhibited significant improvements in reading performance and oculomotor function, whereas no such gains were observed in the control group. The findings support the effectiveness of oculomotor training as a practical approach to enhance reading skills in dyslexic children with eye movement impairments [36].

In summary, an oculomotor training, involving saccadic eye movement tasks, but also other cognitive tasks, has been shown to improve reading and fixation time in dyslexic children, in addition to enhancing complex perceptual motor skill acquisition and improving oculomotor efficiency.

Considering the previous findings, the purpose of the current study was to pilot the efficacy of a pure oculomotor training on visual and cognitive functions in a group of children with reading difficulties.

We expect to obtain an improvement in reading performance, proving the specific role of oculomotor skills in the reading process.

## 2. Materials and Methods

### 2.1. Participants

A total of 21 children participated in this pilot study and were assigned to the experimental group (N = 14, mean age 8.7, SD 1.5, range 7–11, 7 female), or to the control group (N = 7, mean age 8.42, SD 1.65, range 7–12, 3 female) depending on the availability. Groups did not differ in terms of age (U = 56 *p* = 0.61). Participants were recruited among those attending an optometric office for the comprehensive evaluation of learning processes. Inclusion criteria were stereopsis ≤ 120 arcsec., normal binocular vision at cover test, age > 7 years old, at the time of the training, none of the participants met the criteria for SLD and presented a score on the Raven Coloured Progressive Matrices (RCPM) [37] above the 10th percentile [38] but the text reading speed or accuracy are below (<1 SD) the expected values for age and grades. Exclusion criteria were the presence of ocular pathologies, monocular visual acuity < 0.2 LogMAR, and monocular accommodative amplitude less than 2 standard deviations below the mean [39]. A written informed consent was signed by relatives of all participants prior to their participation in the study. The study was conducted according to the guidelines of the Declaration of Helsinki, and it was approved by the ethical committee of the University of Milano Bicocca (0065012/15).

### 2.2. Testing

The testing protocol aimed to measure (a) the baseline performance, (b) the visual changes made by oculomotor training, and (c) to detect the neuropsychological mechanisms underlying the eventual improvement. To this aim, the overall battery covered two main areas of assessment: visual and neuropsychological. Baseline tests comprised the optical refraction to compensate for any refractive error. Experimental testing included: best corrected visual acuity (BCVA), amplitude of accommodation (AA), near point of convergence (NPC), MEM retinoscopy, and, for the oculomotor efficiency, the developmental eye movement (DEM) test. The neuropsychological tests included: text reading (MT2), visual search (Beta Test), the test of Visual Motor Integration (VMI), visual word recognition (Rapid Serial Visual Presentation, RSVP, task), and a computerized evaluation of crowding. A brief description of the adopted tests is outlined below.

#### 2.2.1. Best Corrected Visual Acuity (BCVA)

The Best corrected visual acuity after the subjective refraction was measured with a digital optotype (MOS, Multi Opti System, Dueffe Tecnovision, Pergine Valsugana, TN - Italy https://www.dueffetecnovision.it/ (accessed on 6 October 2025)) at a distance of 4 m using a crowded 3-row presentation [40]. Scoring was performed letter by letter using a logarithmic scale [9,41].

#### 2.2.2. Amplitude of Accommodation (AA)

The amplitude of accommodation was measured monocular using a push-up procedure with a single +0.2 LogMAR symbol (at 40 cm) printed on a stick [39]. The stick was positioned at about 50 cm in front of the patient, and it was moved to the nose at 2 cm/s. A participant was asked to report when he/she perceived the symbol blur. The amplitude of accommodation was calculated in Diopters by the formula:

AA = 1/near point of accommodation (m).

Three measurements were performed for each eye. Since the interest was not in monocular differences, the average of three measurements and RE and LE mean was performed (on Diopters scale).

#### 2.2.3. Near Point of Convergence (NPC)

The NPC was measured binocular using a single +0.2 LogMAR symbol (at 40 cm) printed on a stick. The stick was positioned at about 50 cm in front of the patient, and it was moved to the nose at 2 cm/s. A participant was asked to report when he/she perceived the symbol double. After this point the stick was moved some cm close to the nose and then moved away. In this second part, a participant was asked to report when the two symbols became one [39]. The two points represent the break and recovery of binocular vision. Data were scored in cm.

#### 2.2.4. MEM Retinoscopy

The aim of this procedure was to obtain an objective measurement of Lag of accommodation. The standard procedure of MEM retinoscopy was used [42]. Raw data were compared to subjective refraction, and an equivalent sphere and mean of RE and LE was calculated, obtaining a single value.

#### 2.2.5. Developmental Eye Movement (DEM) Test

The developmental eye movement (DEM) test is a paper-made test aimed at easily assessing and quantifying oculomotor skills in children [43,44]. The DEM test is composed of two parts: vertical and horizontal. The vertical time (VT) is the time spent to name 80 numbers, while the adjusted horizontal time (AHT) represents the time spent to name numbers plus the time to perform eye movements. This score is corrected for errors. The Ratio score represents the ratio between AHT and VT, and it is a measure of eye movement efficiency. The error score represents the number of errors performed in the horizontal part. For the analyses, the raw scores of VT, AHT Ratio, and Errors were used as dependent variables.

#### 2.2.6. Reading

For the assessment of reading abilities, a standard Italian reading test (Prove di lettura MT2) [45] was used. Since participants had different ages and were tested in different periods of the school year, the reading tests were selected, for each participant, according to grade and semester. Data were analysed using reading speed expressed as syll./sec. and accuracy expressed by the number of errors.

#### 2.2.7. Visual Search

A paper and pencil visual search test was used: the Beta test (https://dpg.unipd.it/en/deconelab/materials (accessed on 6 October 2025)). In this test, children were asked to manually cancel the symbol β each time it was detected on the sheet. The target symbol was interleaved by several distractors. The test comprised 155 items with 25 targets, five for each line. The test started with a pre-test in which the task was explained, and it continued with two parts (sheets): the first was low-crowded, and the second was high-crowded, with symbols arranged in a different pattern. Children were asked to manually cancel the symbol β each time it was detected. Execution time and errors were recorded for each condition of crowding.

#### 2.2.8. Visual Motor Integration Test (VMI)–Visual Perception

The visual perception part of the Visual Motor Integration test [46] was applied. Children were required to indicate the exact copy of the target stimuli presented in a series of distractors. The score represents the number of trials correctly performed, considering that the level of difficulty increases over trials in the range of 0–27.

#### 2.2.9. Rapid Serial Visual Presentation

The Rapid Serial Visual Presentation (RSVP) was performed using a custom script built in the Matlab environment. It consists of 4 words composed of four letters (Courier 18pt.) in rapid serial visual presentation with a specific timing. After the presentation, the child has to name the four words. Then, the examiner has to select the correct word spoken from a list. The timing changes trial by trial according to a QUEST modality up to the lower threshold for 75% accuracy [47]. This value represents the score of the task expressed in milliseconds.

#### 2.2.10. Crowding Test

The computerised procedure for determining the crowding threshold was presented by Pelli et al. [48]. The Psychtoolbox Matlab-based procedure requires at the observer to recognise two numbers from the set from 1 to 9 when presented fullscreen in a highly crowded condition in a chessboard matrix. The observer has to name the two numbers without time restrictions. The experimenter enters the corresponding number on the keyboard. The letter size changes trial by trial according to a QUEST procedure to reach the threshold. The presentation was made with a laptop PC (Lenovo Yoga 13“ PC with a FHD screen) from a distance of five metres. Scores were reported in LogMAR scale.

### 2.3. Oculomotor Training

The office-based oculomotor training (OBOT) consists of a training aimed to improve oculomotor behaviour and efficiency, performed five days a week at home (15 min each day) and, once a week, a supervision session in the office (45 min). The procedures were explained to children and to their relatives in an initial individual session. Supervision involves checking the execution of previous exercises, explaining and assigning new exercises, and performing special exercises only in the office. Exercises consist mainly of naming items (numbers, letters, etc.) placed in spaced matrices. The task starts with little matrices (4 × 4) with large numbers printed one next to the other with a large font size. Children were asked to maintain fixation on each item and then move their eyes and name each number row by row. The level of difficulty was increased by modifying:

(1) Spacing between symbols.
(2) Decreasing the size of the items, changing the font and style of characters.
(3) Changing items from number to letter, symbols, syllables, bisyllabic words, bisyllabic pseudowords and lastly, mixed items.
(4) Time, starting without time restriction, followed with the use of a metronome as soon as possible with an increasing rhythm of about from 50 to 120 bpm.
(5) Varying distance: far, intermediate, and near.
(6) Two or four symbols’ matrices can be used simultaneously, naming each letter on each matrix, and varying the distance between them in horizontal, vertical, and longitudinal directions.
(7) Changing scan strategies, from left-to-right to right-to-left, downward to upward, and external to internal items.
(8) Errors were minimised by emphasising accuracy over speed, and the quality of the performance was the main goal before speed. This represents a form of errorless skill acquisition.
(9) The initial level of training was tailor-made to the level of each child.
(10) Items were changed frequently to avoid boredom.

**Figure 1:**
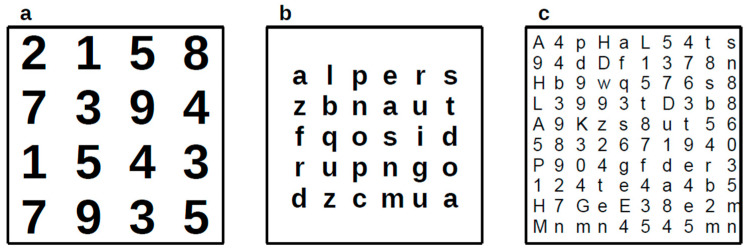
Three examples of matrices with different levels of difficulty used in the oculomotor training. (**a**) a simple 4 × 4 number matrix with large font size. (**b**) 6 × 6 letter matrix with high density and smaller size. (**c**) smaller matrices with mixed symbols and 10 × 10 structure.

Card size ranges between 10 cm × 10 cm, passing through a A4 size (21 cm × 29.7 cm) reaching 50 cm × 50 cm. Some examples of the matrix used are visible in Figure 1.

The OBOT was performed for 6 weeks. To the same extent, the control group was involved in text-reading tasks. Children at home had to read aloud a book for 15 min each day for 6 days a week.

### 2.4. Procedures

Each child was tested and supervised individually in an optometric office. Since most of the visual tests (e.g., visual acuity) are sensitive to the status of optical refraction, to avoid these problems, before testing, a full objective and subjective refraction assessment was performed, firstly using an auto refractometer for the objective refraction, followed by a full subjective refraction evaluation, up to reach the maximum plus to maximum visual acuity lens. The experimental evaluation was performed before and after the training or control condition. During the first examination, two baseline measures were performed: subjective refraction and RCPM. Since the overall evaluation was long (about 2 h), it was run on two separate days.

### 2.5. Statistical Methods

First, a sensitivity analysis was performed to determine the effect size detectable with the sample size tested. Different models of ANOVA were applied for each test, depending on its structure. For BCVA a mixed 3 × 2 × 2 ANOVA was performed with Eye (Right eye, Left eye and Binocular), Session (Pre and Post) as within factors, and Group (Experimental and Control) as between factors. Post-hoc analyses were performed only for BCVA using Holm correction.

Whether not specified, the other analyses were performed using a mixed ANOVA with one within-factor Session with two levels (Pre and Post treatment) and one between-factor Group with two levels (Experimental and Control). Planned contrasts were performed (i) on baseline examinations (Pre) between Groups, and on between Pre and Post examination within the (ii) experimental and (iii) control group. The assumptions of the general linear model were checked by inspecting the Q-Q plots of the residuals, by assessing the homogeneity of variance and evaluating the sphericity using Mauchly’s test. The level of significance alpha was fixed at 0.05. All data are shown in figures as mean ± 1 standard error of the mean (SEM). Statistical analyses and figures were performed using R statistical environment (Version 4.3.3) [49].

## 3. Results

The sensitivity analysis performed for the mixed ANOVA interaction model using the sample size obtained (14 + 7 participants), using an alpha of 0.05, a power of 0.80, and a correlation between repeated measures of 0.7, reveals an effect size detectable of f = 0.21 (equivalent to η^2^_p_ = 0.042).

### 3.1. Best Corrected Visual Acuity

**Figure 2:**
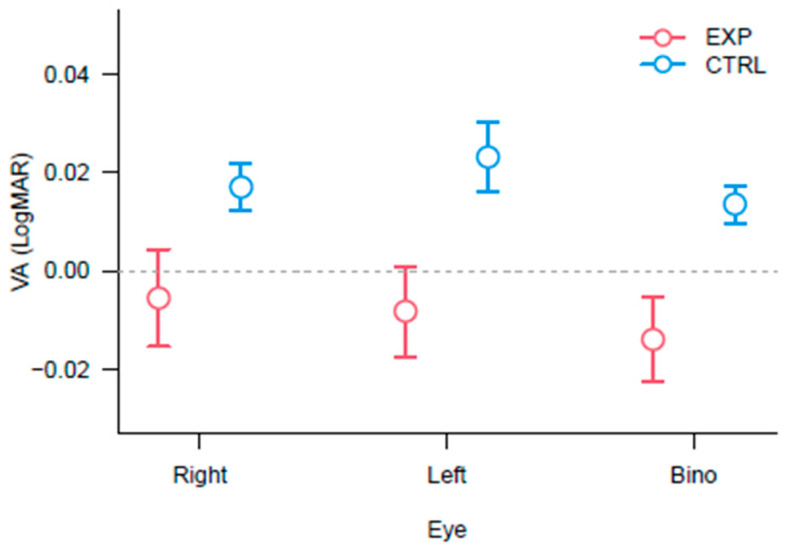
Plot of the BCVA for the participants separated by Group and Eye. The lower the better. The bars represent ±1 standard error of the means (SEM).

To analyse the effect of the training on the BCVA, a mixed 3 × 2 × 2 ANOVA was performed with Eye (Right eye, Left eye and Binocular), Session (Pre and Post) as within factors, and Group (Experimental and Control) as between participants. Results showed a significant main effect of Eye [F_(2,38)_ = 10.86, *p* < 0.0005, η^2^_p_ = 0.36] and a significant main effect of Group [F_(1,19)_ = 4.93, *p* < 0.05, η^2^_p_ = 0.20]. No significant effect of the Session and no interactions were found (see Figure 2). Post-hoc analyses on the factor Eye showed significant differences between OD and OO (*p* < 0.05) and between OS and OO (*p* < 0.0001), showing a better binocular VA than monocular VA. Although the two groups differed in BCVA, with the control group worse than the experimental one, the difference was clinically not relevant (about 1 optotype letter) and under the standard error of measurement [40]. Results are depicted in Figure 2.

### 3.2. Amplitude of Accommodation

**Figure 3:**
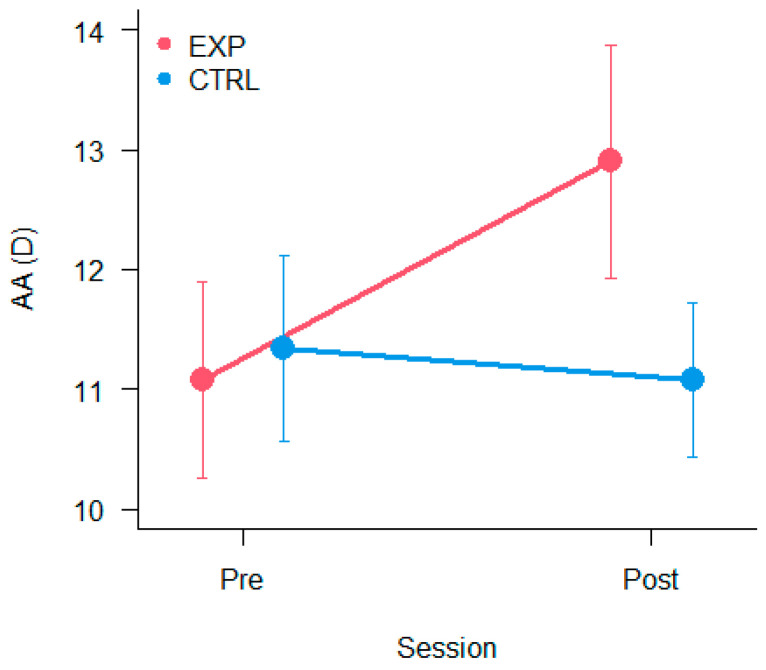
Amplitude of accommodation. The mean values are separated by Group and Session. The bars represent ±1 SEM.

The results of the 2 × 2 ANOVA on amplitude of accommodation showed a significant interaction Session × Group [F_(1,19)_ = 5.10, *p* < 0.05, η^2^_p_ = 0.21]. Planned contrasts showed a significant difference Pre vs Post training only for the Experimental group (*p* < 0.05), which improved in the Post training. Results are depicted in Figure 3.

### 3.3. Near Point of Convergence (NPC)

The analysis of NPC did not show significant results.

### 3.4. MEM Retinoscopy

The analysis of MEM retinoscopy did not show significant results.

### 3.5. Developmental Eye Movement (DEM) Test

**Figure 4:**
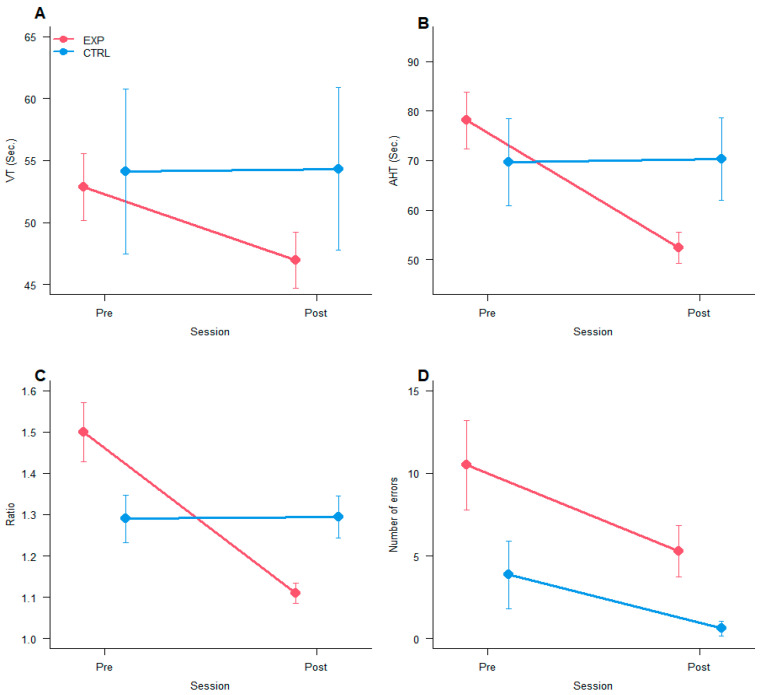
DEM test. Each graph shows the mean results separated by Group and Session for: (**A**) VT; (**B**) AHT; (**C**) Ratio; (**D**) Errors. The bars represent ±1 SEM.

The 2 × 2 mixed ANOVA on each DEM index (VT, AHT, Ratio, Errors) showed the following results. For Vertical Time (VT) there was a significant interaction Group x Session [F_(1,19)_ = 4.2, *p* < 0.05, η^2^_p_ = 0.18], and planned contrast showed a significant difference only for the experimental group between Pre- vs. Post-training (*p* < 0.05, Figure 4A). For adjusted horizontal time (AHT), the results showed a significant main effect of Session [F_(1,19)_ = 14.96, *p* < 0.001, η^2^_p_ = 0.44] and a significant interaction Group x Session [F_(1,19)_ = 16.5, *p* < 0.001, η^2^_p_ = 0.46]. Planned contrasts analyses showed a significant difference only for the experimental group between Pre- vs. Post-training (*p* < 0.0001, Figure 4B). For Ratio (between AHT and VT), results showed a significant main effect of Session [F_(1,19)_ = 12.45, *p* < 0.005, η^2^_p_ = 0.40 and a significant interaction Group x Session [F_(1,19)_ = 13.0, *p* < 0.005, η^2^_p_ = 0.41]. Planned contrasts showed a significant difference only for the experimental group between Pre vs. Post-training (*p* < 0.0001, Figure 4C). All measures, VT, AHT, and Ratio showed a decrease in the experimental group and no differences for the control group in the second assessment compared to the first one. For Errors, results show only a significant effect of *Group* [F_(1,19)_ = 4.71, *p* < 0.05, η^2^_p_ = 0.20, Figure 4D], with more errors for the experimental group than the control group.

### 3.6. Reading

**Figure 5:**
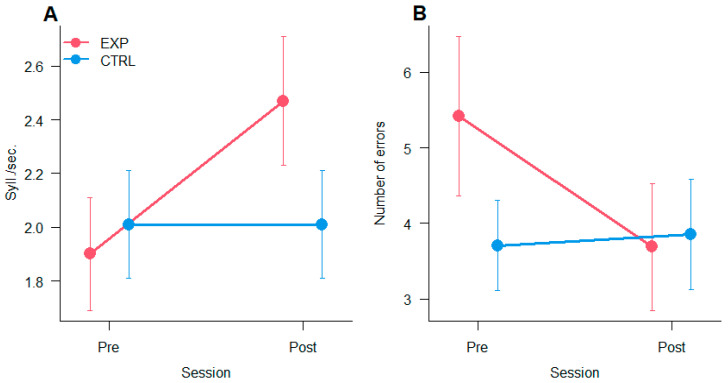
Reading. The two graphs show the reading performance pre and post oculomotor training. (**A**) Speed expressed as Syll/sec. (**B**) Errors. The bars represent ±1 SEM.

The analysis of reading speed measured in syll./sec reports a significant main effect of Session [F_(1,19)_ = 13.89, *p* < 0.005, η^2^_p_ = 0.42] and the interaction Session × Group [F_(1,19)_ = 13.76, *p* < 0.005, η^2^_p_ =0.42] showing an improvement in reading only for the experimental group. The analysis of errors (accuracy) reveals only a significant interaction between Session × Group [F_(1,19)_ = 5.63, *p* < 0.05, η^2^_p_ = 0.24, showing a reduction of errors after training in the experimental group only. Results are depicted in Figure 5A (speed) and Figure 5B (accuracy).

### 3.7. Visual Search

Execution times and errors of the beta visual search were analysed using a two separated mixed 2 × 2 × 2 ANOVA with the factors Crowding (low and high), and Session (Pre and Post) as within factors and Group (Experimental and Control) as between factors. The analysis showed no significant results for both execution times and errors.

### 3.8. Visual Motor Integration Test (VMI)–Visual Perception

**Figure 6:**
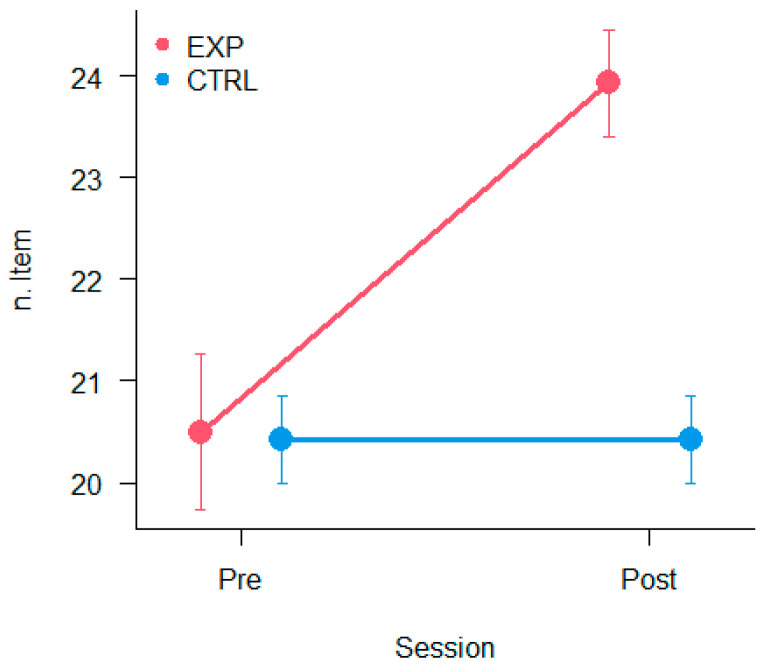
Mean results on VMI–visual perceptual test. The dependent variable was the number of items recognised correctly. The bars represent ±1 SEM.

Using a mixed 2 × 2 ANOVA with Session (Pre and Post) and Group (Experimental and Control) on VMI score, the results showed a significant main effect of Session [F_(1,19)_ = 18.15, *p* < 0.0005, η^2^_p_ = 0.49], and a Session × Group significant interaction [F_(1,19)_ = 18.15, *p* < 0.0005, η^2^_p_ = 0.49]. Planned contrasts showed a significant improvement only for the experimental group between Pre- vs. Post-training (*p* < 0.0001). This result was represented on Figure 6.

### 3.9. Rapid Serial Visual Presentation

Results showed a significant main effect of Session [F_(1,19)_ = 7.71, *p* < 0.05, η^2^_p_ = 0.30]. The time needed to name words decreased from 1757 ms (SD 995, first session) to 1296 ms (SD 630, second session). No significant effect of Group and Session, and no interactions were found.

### 3.10. Crowding Test

**Figure 7:**
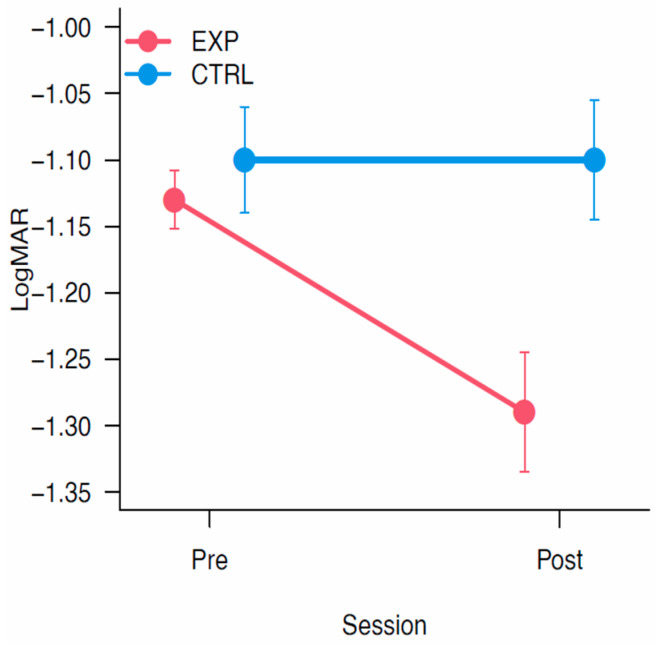
Crowding. The graph report the results on the crowding test. Data were reported in LogMAR, the lower the better. The bars represent ±1 SEM.

A mixed ANOVA showed a main effect of Session [F_(1,17)_ = 6.52, *p* < 0.05, η^2^_p_ = 0.28], a significant main effect of Group [F_(1,17)_ = 5.66, *p* < 0.05, η^2^_p_ = 0.25] and a significant interaction Session × Group [F_(1,17)_ = 6.75, *p* < 0.05, η^2^_p_ =0.28]. Planned contrasts showed a significant improvement only for the experimental group between Pre and Post-training (*p* < 0.005). Results are depicted in Figure 7.

## 4. Discussion

The aim of this pilot study was to explore the effect of the office-based oculomotor training on visual and reading skills in a group of children with learning difficulties. The main results show an improvement, specific to children involved in the oculomotor training, in some abilities. Specifically, eye movement skills, reading, and some cognitive functions have been improved through the systematic use of various charts of symbols in structured training. Charts included letters, numbers, symbols, etc. Using different charts, training sessions have been kept engaging and challenging, which led to comprehensive improvements in oculomotor abilities.

With regards to visual acuity (VA), at baseline, with the best optical correction, a difference in BCVA was found. The mean difference between groups (about one letter) was statistically significant but just slightly over the minimum level of detectability using a chart optotype [40]. Moreover, VA improvement after OBOT was not expected (and not found), making this difference of limited relevance.

When considering the functional aspects of vision, specific improvements after oculomotor training were detected only in the accommodation amplitude. It is worth noting, first, that the OBOT was not focused on accommodation capacity [50] and, second, that the improvement occurred in children with a close to normal amplitude of accommodation, without any remarkable deficit. Both training and control conditions (OBOT and reading) are tasks performed at near distance, this may induce a stimulation on the accommodative system. Conversely, the specific improvement in the experimental group may be ascribed to OBOT because the accommodative demands may vary from the charts used at different distance compared to a fixed distance demand during reading. In addition, the OBOT instruction to fixate with a high degree of attention during training (e.g., “keep your eyes focused on a specific letter”), may contribute to the precise accommodative demand, while reading does not require precise requirement of accommodation [51]. Conversely, no improvements were found in the response of MEM retinoscopy and in the total amplitude of convergence (NPC). Other training specifically targeted for binocular and accommodative visual abilities (office-based vergence and accommodative training), in contrast, found improvements in NPC [52]. That kind of training used specific exercises aimed to enhance these skills; consequently, the improvement of NPC is an expected improvement.

Considering the main, although indirect measure of oculomotor abilities (DEM), significant and specific improvements only in the experimental group were found after training in the horizontal, vertical, and ratio components of this test. This is the main improvement found, although well expected since the oculomotor training performed was relatively similar to the test itself. Overall, the results of OBOT obtained in children with learning difficulties on the DEM test showed specific improvement in oculomotor abilities when these abilities were partially compromised; the DEM test resulted, thus, adequate to detect initial deficits and the following improvements [53]. Concerning accuracy, the analyses showed only a group effect, which was due to a baseline difference, with the experimental group making more errors than the control group. Overall, the number of errors was small, and no significant reduction was observed. Furthermore, no learning effect (shorter time in the second evaluation) was observed on the DEM test in the control group, simply involved in reading aloud sessions. DEM has been shown to have moderate learning effects and test-retest reliability, which limits its use as a time series or pre/post-treatment evaluation tool [54]. In the present study, however, the time between test and retest was longer (six weeks) than that used before (two weeks), and participants were children with learning difficulties rather than typical school-aged children. Probably, the short-term learning effects found over two weeks disappeared after six weeks. A previous study on dyslexic children found that reading-like oculomotor exercises included in a computerised training composed of 4 different tasks, had significantly reduced fixation time during reading [33]. A study on dyslexic children evaluated the effectiveness of oculomotor training (saccades, pursuits, fixation): based on an eye tracker evaluation, all pure oculomotor parameters (pursuit gain, saccade latency, velocity, and accuracy) were improved overall [21].

The DEM test has already been used in the evaluation of rehabilitation before and after an oculomotor treatment, but in adult stroke patients [55], demonstrating the effectiveness of the training as well as the validity of the DEM test as a clinical test of saccadic tracking performance in stroke patients.

The main result of this pilot study is that reading, both in terms of speed and accuracy, improved after the oculomotor training. It may be argued that the same reading test was administered in both sessions to each child, and a learning effect may cause this improvement. However, since the improvement did not occur in the control group, it is plausible to assume that the reading benefits may be explicitly attributed to the oculomotor training, and no learning effect was found (as described for DEM test). Maintaining attention to each letter and performing specific saccades symbol to symbol have indirectly improved the reading process. Although the scan path of the training exercises seems to be relatively similar to the reading one, the two materials are very different. Moreover, during training, different scan directions were used, not only left to right. The present results suggest that the training was generalised to the reading process. Indeed, our results are in line with previous evidence in favour of the beneficial effect of oculomotor exercises on reading performance in dyslexic children [33].

Not all measures improved after the oculomotor training, suggesting specific benefits other than general ones. In particular, there were changes neither in the RSVP procedure, which is a psychophysical assessment of reading without eye movements, nor in visual search abilities. Conversely, perceptual skills and the ability to resolve crowding showed specific improvement only after training. Taken together, these results imply that changes have occurred in specific cognitive abilities but not in others. Text reading has improved, but RSVP reading has not. This implies that eye movements help to place stimuli in the fovea, which, in addition to having greater acuity, has also greater focal attention [56]. There is a possibility that training does not improve focal attention but only the ability to orient to stimuli more quickly and easily. This would explain why in a condition like RSVP, in which eye movements are not allowed, the performance does not improve, but in reading, in which oculomotor behaviour is largely involved, we detect improvement. The training of saccades could facilitate the control of orientation of attention, inhibitory functions, and flexibility, which are all closely related to the cognitive abilities required to explore text [33,57].

Crowding has been shown to be implicated in developmental [7] and acquired [58,59] dyslexia, and the ability to resolve crowding, measured with a specific psychophysical procedure, may be called into question to account for the observed improvement in reading, eye movements, and perception. The effect of training on visual perceptual skills was, indeed, confirmed by Dalvand et al. [60] in a study where online video games were used to improve eye movements and, indirectly, perception.

In the present study, children were required to name visual symbols during eye movement treatment. As a result, they trained phonological skills during oculomotor training as well. Even though this is realistically true, and the DEM VT score supports this theory, no significant improvements were found in a psychophysical measure of reading without eye movements, making this explanation less likely.

In a previous study investigating the effect of oculomotor training on dyslexic children’s performance, Jafarlou et al. [34] adopted three subtests of the Cambridge Neuropsychological Test Automated Battery (CANTAB): internal extra-dimensional set shift (IED), rapid visual information processing (RVP) and spatial working memory (SWM). They reported that performance in all three subtests (and functions) improved after oculomotor training.

Using a different point of view, training focused on the visual efficiency of the accommodative and vergence system of the eyes (Office Based Vergence Accommodative Treatment) was effective to treat convergence insufficiency [52] but had not (indirect) effect on the reading skills of children [61].

This oculomotor training is part of a series of innovative approaches aimed at remediating dyslexia by targeting underlying specific deficits that may contribute to reading difficulties [62,63].

It is worth noting that, as a pilot study, we acknowledge certain limitations, including the relatively lower sensitivity of visual assessments using a simple paper-based oculomotor test (DEM) compared with advanced eye-tracking systems.

An eye-tracking study with a bigger sample size is needed to confirm these results. In particular, future studies may be directed to investigate: (i) the effectiveness of the OBOT training, using an eye-tracking technology to evaluate the oculomotor performance, (ii) the specificity of OBOT on some cognitive tests targeted to measure naming and perceptual abilities, (iii) the maintenance of improvement by adding a follow-up evaluation and, most importantly, (iv) a RCT study in a large group of children other than specific instruments and tests [40,64]. In particular, the most critical limitation is the small sample size. As a pilot investigation, the primary aim of this study was to evaluate the effectiveness of the training on reading and related cognitive mechanisms, while also providing a reliable estimate of the effect size to guide future randomized controlled trials on the same intervention. The limited sample size should be interpreted in light of the inherent challenges in recruiting and retaining control participants.

## 5. Conclusions

The results of this study show that OBOT training improves not only oculomotor abilities but also reading skills and visuo-perceptual skills. These primary findings suggest that improving oculomotor abilities may indirectly contribute to improving reading skills in children with learning difficulties.

## Data Availability

The data presented in this study are available on request from the corresponding author. The data are not publicly available due to restrictions included in the informed consent provided by participants.

## Abbreviations

The following abbreviations are used in this manuscript:

AA	Amplitude of Accommodation
BCVA	Best Corrected Visual Acuity
DD	Developmental Dyslexia
DEM	Developmental Eye Movement Test
NPC	Near Point of Convergence
OBOT	Office Based Oculomotor Training
RSVP	Rapid Serial Visual Presentation
VA	Visual Acuity
VMI	test of Visual Motor Integration
